# Exploring the Heat Shock Transcription Factor (*HSF*) Gene Family in Ginger: A Genome-Wide Investigation on Evolution, Expression Profiling, and Response to Developmental and Abiotic Stresses

**DOI:** 10.3390/plants12162999

**Published:** 2023-08-20

**Authors:** Dongzhu Jiang, Maoqin Xia, Haitao Xing, Min Gong, Yajun Jiang, Huanfang Liu, Hong-Lei Li

**Affiliations:** 1 College of Landscape Architecture and Life Science, Chongqing University of Arts and Sciences, Chongqing 402160, China; jiangdongzhu11@163.com (D.J.); xiamq@cqwu.edu.cn (M.X.); htxing205@126.com (H.X.); jiangyajun228@163.com (Y.J.); 2 College of Horticulture and Gardening, Yangtze University, Jingzhou 433200, China; 3 College of Biology and Food Engineering, Chongqing Three Gorges University, Chongqing 404100, China; gongmin1999@163.com; 4 Key Laboratory of Plant Resources Conservation and Sustainable Utilization, South China Botanical Garden, Chinese Academy of Sciences, Guangzhou 510650, China; hfliu@scbg.ac.cn

**Keywords:** ginger, *HSF* gene family, abiotic stress, expression patterns

## Abstract

Ginger is a valuable crop known for its nutritional, seasoning, and health benefits. However, abiotic stresses, such as high temperature and drought, can adversely affect its growth and development. Heat shock transcription factors (*HSFs*) have been recognized as crucial elements for enhancing heat and drought resistance in plants. Nevertheless, no previous study has investigated the *HSF* gene family in ginger. In this research, a total of 25 *ZoHSF* members were identified in the ginger genome, which were unevenly distributed across ten chromosomes. The *ZoHSF* members were divided into three groups (HSFA, HSFB, and HSFC) based on their gene structure, protein motifs, and phylogenetic relationships with Arabidopsis. Interestingly, we found more collinear gene pairs between *ZoHSF* and *HSF* genes from monocots, such as rice, wheat, and banana, than dicots like *Arabidopsis thaliana*. Additionally, we identified 12 *ZoHSF* genes that likely arose from duplication events. Promoter analysis revealed that the hormone response elements (MEJA-responsiveness and abscisic acid responsiveness) were dominant among the various cis-elements related to the abiotic stress response in *ZoHSF* promoters. Expression pattern analysis confirmed differential expression of *ZoHSF* members across different tissues, with most showing responsiveness to heat and drought stress. This study lays the foundation for further investigations into the functional role of *ZoHSFs* in regulating abiotic stress responses in ginger.

## 1. Introduction

High temperatures have a detrimental effect on plant growth, development, and metabolism, significantly impacting crop yield and quality [1,2,3]. Heat shock transcription factors (HSFs) are crucial proteins involved in regulating plant responses to high temperatures. [4,5]. Previous studies have shown that *HSFs* possess the ability to enhance plant resistance to high temperatures by engaging with heat shock elements and subsequently activating the expression of downstream genes [6,7,8]. A typical HSF protein contains five conserved oligomerization domains [9]: a DNA-binding domain (DBD), an oligomerization domain (OD) [10,11], a nuclear localization signal domain (NLS), a nuclear export signal domain (NES) [4,12], and a C-terminal activation domain (CTAD) [13,14]. It is worth noting that the CTAD region is only present in certain members of the *HSF* gene family. However, it plays a crucial role in self-activation by *HSFs* [15]. Based on the characteristics of their oligomerization structures, the *HSF* gene family can be divided into three branches: A, B, and C [11].

Ever since the first *HSF* gene was discovered in yeast, researchers have identified an increasing number of *HSFs* in various plant species, including *Arabidopsis thaliana* [16], rice (*Oryza sativa*) [11], potato (*Solanum tuberosum*) [17], tomato (*Solanum lycopersicum*) [18], wheat (*Triticum aestivum*) [19], and maize (*Zea mays*) [14]. The respective species possess 21, 25, 27, 24, 82, and 25 *HSF* genes. Overexpression of *HSFA9* in tobacco has been shown to enhance the accumulation of carotenoids, chlorophyll, and anthocyanins [20]. It also promotes the expression of genes involved in light morphogenesis (PHYA, PHYB, and HY5), leading to improved photosynthesis and tobacco growth. The modulation of heat shock proteins like HSP70 and HSP90 by *HSFs* is involved in regulating flowering time and vernalization pathways, thereby suppressing the expression of FLC and promoting flowering [21]. Moreover, the overexpression of the *HaHSFA9* gene in tobacco seeds benefits sunflower seeds by enhancing protein accumulation during embryogenesis and promoting callus growth [22].

Additionally, *HSF* transcription factors are crucial in plants’ responses to various abiotic stresses, including heat, oxidative stress, and salt stress. Elevating the levels of *HsfA3s* in Arabidopsis enhances heat resistance but may increase susceptibility to salt due to changes in proline breakdown pathways [23]. Overexpression of *HsfA2* in Arabidopsis leads to the early activation of target genes and improves tolerance to hypoxia [24]. The *AtHSFA6b* gene is significantly upregulated under high salt and drought conditions, contributing to drought, salt, and heat stress responses mediated by ABA [25]. Moreover, *HSFs* regulate the expression of other stress response-related genes, influencing plant adaptability. For example, *ZmHsf11* negatively regulates oxidative stress-related genes, reducing plant tolerance to heat stress by increasing ROS levels and reducing proline content [26]. *HSFA2* enhances the heat tolerance in Arabidopsis by promoting the expression of heat-responsive genes, such as APX2 and HSP [27]. In the case of tomatoes, *HsfA3* has been observed to interact with *MAP* kinases, playing a role in regulating the heat stress response [28]. Additionally, *HSFs* have been found to participate in somatic embryo maturation and interact with glutathione metabolism [29], ABA signaling [30], and secondary metabolites [31], contributing to enhanced thermotolerance in plants.

Ginger (*Zingiber officinale*) is a perennial herb belonging to the Zingiberaceae family, renowned for its distinct aroma and strong taste. It serves not only as a valuable medicinal resource due to its high gingerol content but also contains numerous active compounds that possess beneficial properties, including blood pressure regulation, lipid reduction, antioxidation, and immune enhancement [32,33]. Nevertheless, abiotic factors, like high temperature and drought, negatively impact the normal growth and development of ginger, leading to reduced yield and compromised quality [34,35,36]. While *HSFs* play a crucial role in various abiotic stresses, no reports have documented the presence of *HSF* gene family members in ginger.

In our previous investigation, we accomplished the successful sequencing and chromosomal assembly of the ginger genome. This achievement has provided a solid foundation for conducting an all-inclusive examination of the *HSF* gene family in ginger [37]. The current study represents the first identification of 25 members of the *HSF* gene family from the ginger genome sequence. We analyzed various characteristics of these genes, including gene structures, promoter cis-acting elements, chromosomal locations, conserved motifs, collinearity, replication events, and expression patterns. We further elucidated the evolutionary relationships between ginger and other plant species, such as *Arabidopsis thaliana*, *Triticum aestivum*, *Oryza sativa*, and *Musa acuminata*. Additionally, we examined the expression profiles of the *HSF* genes under high temperature stress. Through our bioinformatic analysis, we gained important insights into the *ZoHSFs* and provided valuable information for further investigations into the role of *HSF* family genes in ginger’s response to abiotic stress.

## 2. Results

### 2.1. Genome-Wide Identification of the HSF Family in Ginger

We used the hidden Markov model (HMM) method with the HSF protein domain (PF00447) as the query to identify a total of 25 *HSF* gene sequences in the *Z. officinale* reference genome. A variety of gene characteristics have been provided, including protein molecular weights (MW), isoelectric points (pI), and coding sequence (CDS) lengths (Table 1). Within the set of 25 *HSF* genes, *ZoHSF14* is identified as the shortest, encompassing a length of 239 amino acids (aa). Conversely, *ZoHSF07* is distinguished as the longest among the group, spanning a length of 585 aa. The MW of the proteins ranged from 27.65 kDa (*ZoHSF14*) to 73.07 kDa (*ZoHSF07*), and the pI ranged from 4.62 for *ZoHSF17* to 9.62 for *ZoHSF02* (Table 1).

### 2.2. Chromosomal Distribution and Classification of the ZoHSF Genes

**Figure 1:**
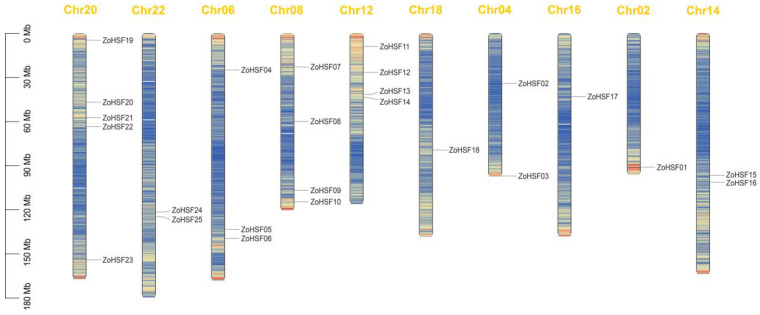
Chromosomal location of *HSF* members in ginger. The red color indicates a higher gene density in this chromosome region, while blue represents a lower gene density.

We found that the 25 identified protein sequences were unevenly located on ten chromosomes of *Z. officinale*, excluding chromosome 10. To prevent confusion and enhance clarity, we have renamed these sequences from *ZoHSF01* to *ZoHSF25* based on their specific chromosomal locations. Each chromosome containing the *ZoHSF* genes varies in the number of genes present. Chromosome 20 had the highest number of *ZoHSF* genes (five), followed by chromosomes 02 and 08 with four genes each. Whereas chromosomes 2, 18, and 16 each had only one *ZoHSF* gene (Figure 1).

**Figure 2:**
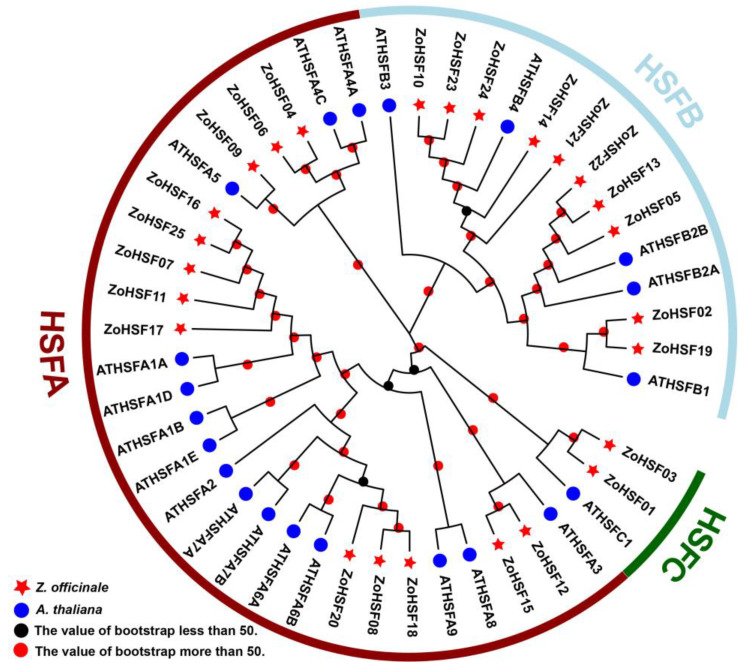
The phylogenetic tree illustrates the evolutionary relationships between *HSF* genes of ginger and Arabidopsis. Three distinct color-coded groups are depicted, representing HSFA, HSFB, and HSFC.

In order to examine the categorization of *ZoHSF* genes, we generated a phylogenetic tree that included *A. thaliana* with 21 *HSF* genes and ginger with 25 *HSF* genes. Utilizing distinctions observed within the HR-A/B domain and phylogenetic connections within the *ZoHSF* genes, we classified the *ZoHSF* members into three major groups: HSFA, HSFB, and HSFC (Figure 2). Out of the three groups, group A consisted of the highest number of *ZoHSF* members (13), followed by group B (10), with group C exhibiting the fewest members (2).

### 2.3. Gene Structure, Motif Composition

**Figure 3:**
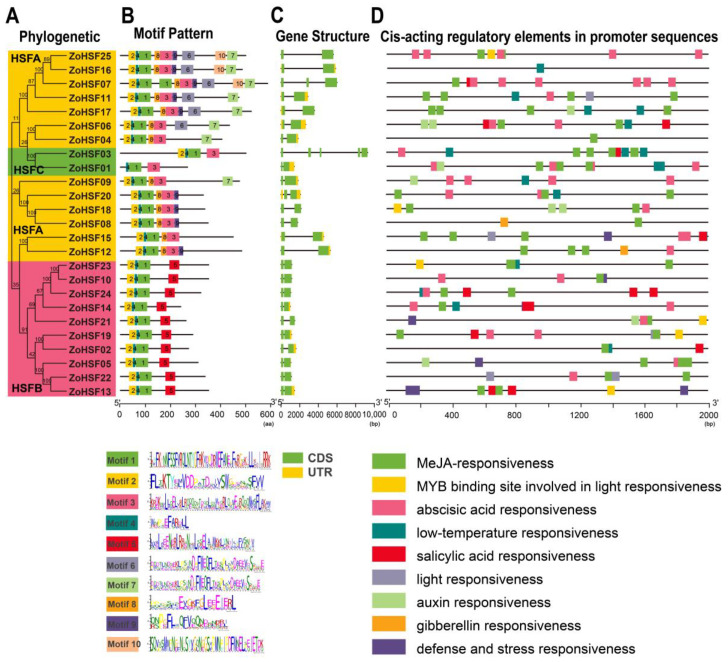
Phylogenetic relationships, gene structures, architecture of the conserved protein motifs, and the cis-acting elements analysis of the *ZoHSFs*. (**A**) The phylogenetic tree was constructed based on the full-length sequences of ginger HSF proteins, including HSFA, HSFB, and HSFC. (**B**) Motif patterns of ZoHsf genes. Motifs numbered 1 to 10 are visually represented by distinct colored boxes. The detailed sequence information for each motif can be found in additional file 1 (Table S1 and Table S2). (**C**) Exon–intron structures of *ZoHSF* genes. (**D**) The cis-acting elements of the *ZoHSF* promoter region, and different color blocks represent different elements.

To investigate the *ZoHSF* gene’s structural composition, we conducted a thorough analysis of exon and intron numbers and distribution. With the exception of *ZoHSF03*, which contains five exons, the remaining 24 members of the *ZoHSF* genes possess two exons each (Figure 3C). The *ZoHSF*s displayed a conservation of the number of exons, although the locations of the exons varied. Similarities were observed in the number and length of exons in both the HSFA and HSFB subgroups, both containing two exons. However, in the HSFC, there was a significant disparity between *ZoHSF03*, which had more than twice the number of exons as compared to *ZoHSF01*, indicating a notable difference (Figure 3C).

To analyze the characteristic regions of the *ZoHSF* genes, we utilized the online MEME tool to examine the motifs of the *ZoHSF* genes. Based on the results obtained from MEME, we constructed a schematic diagram representing the structures of the *ZoHSF* genes and the sequence of 10 motifs. Among the 24 members of the *ZoHSF* family, motifs 1, 2, and 4 were present, while *ZoHSF03* only displayed motifs 1 and 4 (Figure 3B). It is noteworthy that motif 5 was exclusively found in the HSFB, while motifs 6, 7, 8, 9, and 10 were solely detected in HSFA. All members of HSFA possessed motifs 1, 2, 3, 4, and 8 (Figure 3B). Similarly, the motifs present in HSFB members were consistently comprised of motifs 1, 2, 4, and 5. In terms of the HSFC members (*ZoHSF01* and *ZoHSF03*), they exclusively contained motifs 1, 3, and 4. Generally, each subgroup of *ZoHSF* members exhibited similar motifs, but there were substantial differences between different subgroups.

### 2.4. Cis-element Analysis of ZoHSFs

To investigate the regulatory mechanisms of *ZoHSF* genes in abiotic stress responses, we extracted the upstream 2 kb sequences of 25 *ZoHSF* genes for cis-acting element analysis. Our analysis revealed the presence of multiple cis-acting elements in the *ZoHSFs*, including environmental response elements and hormone response elements. The diversity of these cis-acting elements suggests that different *ZoHSF* genes may have distinct potential functions. Most of the promoters of *ZoHSF* genes contain hormone-responsive elements, such as abscisic acid responsiveness (ABRE), gibberellin responsiveness (GARE-motif, P-box, and TATC-box), salicylic acid responsiveness (TCA-element and SARE), auxin responsiveness (TGA-element, TGA-box, AuxRR-core), and MEJA-responsiveness (CGTCA-motif and TGACG-motif) (Figure 3D). Of these, MEJA-responsiveness and abscisic acid responsiveness were commonly observed in the promoter region of *ZoHSF* genes, with 23 *ZoHSF* genes displaying MEJA responsiveness and 18 *ZoHSF* genes displaying abscisic acid responsiveness (Figure 3D). Only two genes (*ZoHSF08* and *ZoHSF12*) contained gibberellin responsiveness (Figure 3D). Furthermore, the promoter region of *ZoHSF* genes also exhibited stress responses in the MYB transcription factor binding site (MBS), low temperature responsiveness (LTR), and light responsiveness, suggesting their involvement in different stress responses. Overall, our findings suggest that *ZoHSF* genes have the ability to participate in various abiotic stress responses and respond to a wide range of hormones.

### 2.5. Evolutionary relationships of the ZoHSFs

**Figure 4:**
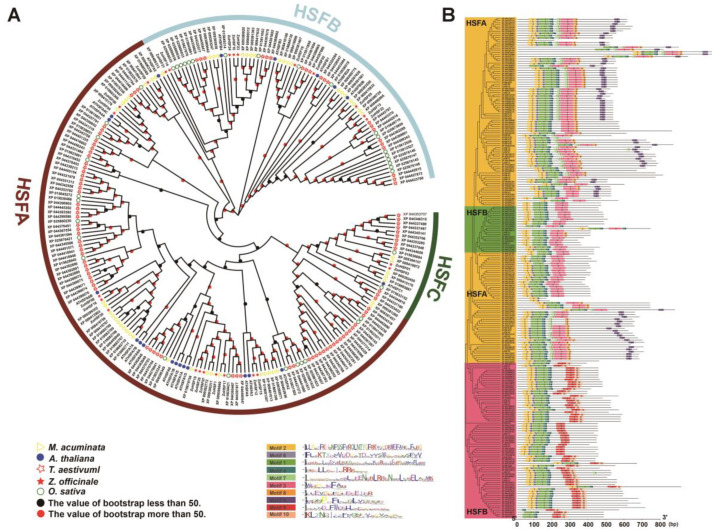
Phylogenetic relationships and motif compositions of the HSF proteins from five different plant species. (**A**) Phylogenetic relationships of the HSF proteins from five different plant species. (**B**) Motif compositions of the HSF proteins from five different plant species.

To investigate the evolutionary relationships between the *ZoHSF* genes, an analysis was conducted using MEGA 5.0. The phylogenetic tree was constructed using representative HSF protein sequences from five different species: *O. sativa*, *M. acuminata*, *Z. officinale*, *T. aestivum* (monocotyledonous plants), and *Arabidopsis* (dicotyledonous plants). The clustering method based on the *HSF* of Arabidopsis divided the *HSF* members into three main groups: HSFA, HSFB, and HSFC (Figure 4). Group A proteins were found to contain a C-terminal initiation motif (AHA), while the oligomerization motif (HR-A/B) in group B proteins had seven fewer bases as compared to group C proteins. It is interesting to note that each subgroup included *HSF* family members from all five species, suggesting that the differentiation time of these five species was later than that of *HSF* transcription factors. As demonstrated in Figure 3B, motif analysis revealed the presence of 10 specific motifs among the *HSF* members from the five species. Almost all members had motif 1, motif 2, and motif 4. Gene structure analysis indicated that most *HSF* members had two exons, which is consistent with the findings in *ZoHSFs*. The results of the evolutionary and motif analyses indicated that *HSF* from the same subgroup in different species often had similar motif compositions, suggesting potential functional similarities between these proteins.

### 2.6. Genome-Wide Replication Events and Synteny Analysis of ZoHSFs

**Figure 5:**
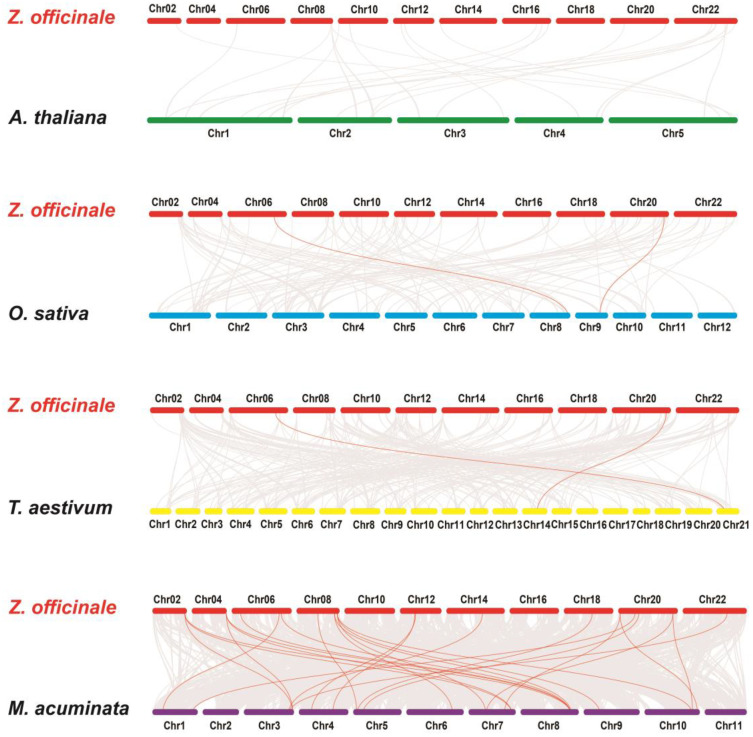
Synteny analysis between the *HSF* genes of ginger and four representative plant species.

To investigate the phylogenetic relationship of *ZoHSFs* in ginger, we compared them with those from four other species: *O. sativa*, *M. acuminata*, *Arabidopsis*, and *T. aestivum*. The results, shown in Figure 5, indicate collinear relationships between ginger and these species. Gray lines represent all genes with collinear relationships, while red lines represent *HSF* genes. Among the analyzed species, ginger exhibited syntenic relationships with 17 *ZoHSF* genes in banana, followed by rice (2), wheat (2), and Arabidopsis (0). The number of collinear gene pairs between *ZoHSFs* and banana, rice, wheat, and Arabidopsis were identified as 26, 2, 2, and 0, respectively. Notably, two *ZoHSF* genes (*ZoHSF01* and *ZoHSF23*) in ginger showed collinearity with *HSF* genes in three species: *O. sativa*, *M. acuminata*, and *T. aestivum*. This suggests that these two *ZoHSF* genes may have existed prior to the differentiation of these species.

**Figure 6:**
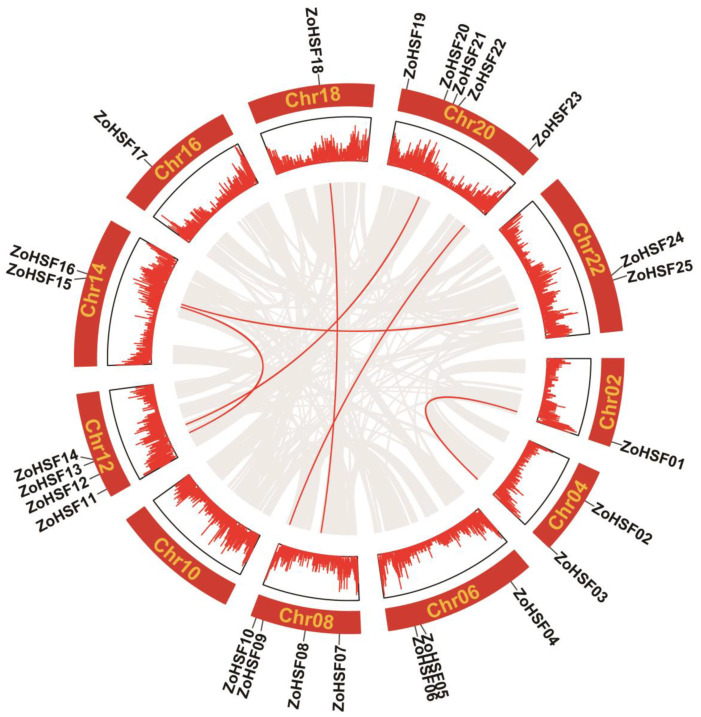
Schematic presentations of the inter-chromosomal relationships of ginger *HSF* genes. The red lines indicate duplicated *HSF* gene pairs in ginger. The chromosome number is indicated in the middle of each chromosome.

To examine the genome-wide replication events of *ZoHSF* members in ginger, the software McScan X was employed. The analysis results, depicted in Figure 6, indicated a collinear pattern among duplicated gene pairs throughout the ginger genome. The *ZoHSF* genes was marked with a red line, while the grey line represented all duplicated gene pairs. This analysis revealed that a total of 12 *ZoHSF* genes were identified as part of six replicated gene pairs. This suggests that these genes may have been generated through genome-wide replication events, highlighting the significant role of such events in the expansion of the *ZoHSF* gene family.

### 2.7. Expression Patterns of ZoHSF Genes in Different Plant Tissues and Various Abiotic Stresses

**Figure 7:**
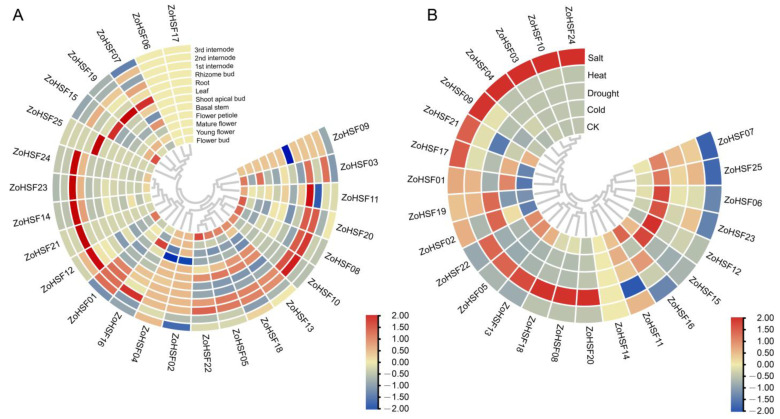
Expression profiles of the ginger *HSF* genes. (**A**) Hierarchical clustering of expression profiles of ginger *HSF* genes in 12 samples, including different tissues and developmental stages. (**B**) Expression profiles of *HSF* genes under abiotic stress treatments.

To explore the potential functions of the *ZoHSF* genes in different developmental stages of ginger organs/tissues and various abiotic stresses, we used RNA-seq data to detect their expression patterns. In different plant tissues, the expression of *ZoHSF* family members is significantly different (Figure 7A). Most genes were detected in the all kinds of tissues, such as *ZoHSF02*, *ZoHSF05*, and *ZoHSF18*, while some genes were not detected in these three tissues, such as *ZoHSF06* and *ZoHSF17*. The genes that are not expressed in all tissues may be a pseudogene or have a special temporal or spatial expression pattern that is not detected in our data. Further studies showed that the expression patterns of individual *ZoHSF* genes were tissue-specific. For example, *ZoHSF07*, *ZoHSF19*, and *ZoHSF15* were only highly expressed in ginger leaves; *ZoHSF21* and *ZoHSF12* were only expressed in ginger stems; and *ZoHSF25* was only highly expressed in ginger roots. The expression of some genes showed significant trends at different developmental stages. For example, the expression level of *ZoHSF18*, *ZoHSF05*, *ZoHSF22*, and *ZoHSF13* decreased first and then increased with flower development, while the expression level of *ZoHSF21* and *ZoHSF07* increased first and then decreased with flower development (Figure 7A).

To investigate the potential functions of the *ZoHSF* genes under various abiotic stresses, we used RNA-seq data to detect their expression levels under cold, heat, drought, and salt treatments; *ZoHSF14* were not expressed in any of the four treatments. The other 24 *ZoHSF* genes were induced by at least one stress treatment. Among of them, the expression of 9 genes was induced by cold, that of 11 genes by heat, that of 7 genes by drought, and that of 16 genes by salt (Figure 7B). Therefore, the greatest number of these genes was induced by salt and the lowest by drought.

### 2.8. Expression Patterns of ZoHSFs under High Temperature and Strong Light Stress

**Figure 8:**
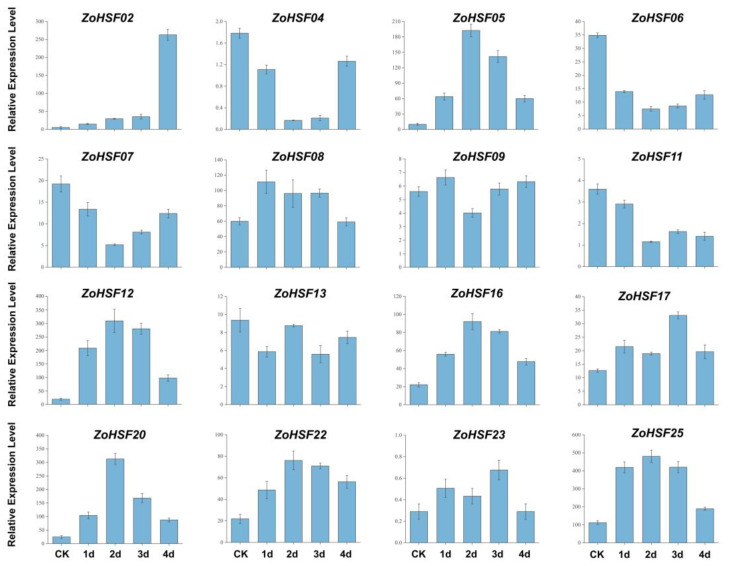
Expression analysis of *HSF* genes under abiotic stresses by qRT–PCR. Data were normalized to *TUB-2* gene, and vertical bars indicate standard deviation.

The expression pattern of ginger *ZoHSF* members under natural high temperature and strong light stress were analyzed using qRT–PCR (Figure 8). Under high temperature and strong light stress, the expression levels of some *ZoHSF* genes showed a trend of decreasing first and then increasing, including *ZoHSF04*, *ZoHSF06*, and *ZoHSF07.* Some *ZoHSF* genes can be induced by high temperature and strong light, showing a trend of increasing first and then decreasing, such as *ZoHSF05*, *ZoHSF12*, *ZoHSF16*, and *ZoHSF25*. The expression levels of *ZoHSF05*, *ZoHSF12*, *ZoHSF23*, and *ZoHSF20* reached the highest peak after 3 days of treatment under natural high temperature and a strong light environment, respectively. The expression patterns of these *ZoHSF* genes under drought treatment were time-specific, and their expression levels peaked after 3 days of treatment and then decreased after 4 days of treatment.

## 3. Discussion

Ginger (*Zingiber officinale*) is widely acknowledged for its economic importance. However, it is susceptible to environmental stresses, such as high temperatures, which can have detrimental effects on its productivity [38]. Previous studies have provided evidence suggesting that heat shock transcription factors (*HSFs*) play a crucial role in regulating plant responses to different biotic stresses, including high temperature and drought conditions [39]. While genome-wide analyses of *HSF* gene families have been conducted in various species with sequenced genomes, there has been no specific investigation into *HSF* genes in ginger. This study aims to bridge the research gap by identifying the *HSF* members of ginger at the genomic level.

The number of *HSF* gene family members varies among different species. For instance, *Arabidopsis thaliana* has 21 *HSF* members [40], goatgrass (*Aegilops tauschii*) has 19 members [41], maize (*Zea mays*) has 25 members [15], and *Triticum urartu* has 17 members [37]. Previous research has indicated that gene family size can change due to genome recombination and expansion [42]. Throughout the evolution of angiosperms, genome duplications frequently take place, which subsequently contribute to the amplification of gene families [43]. In this study, we identified and classified 25 *HSF* genes from the ginger genome and designated them as *ZoHSFs*. They were found to be unevenly distributed over ten chromosomes and classified into three distinct groups (A, B and C) based on phylogenetic analysis. The distribution of *HSF* gene families in ginger followed a similar pattern to other plant species, with a higher number of *HSF* genes in groups A and B as compared to group C. This suggests that homologous genes with similar motifs and arrangement may exhibit functional redundancy, while heterologous genes may have similar functions. Furthermore, when comparing the *HSF* gene family of ginger to Arabidopsis, it was observed that the number of *ZoHSFs* in groups B and C exceeded that of *AtHSF* members. This finding suggests that after the early ancestor differentiation of ginger and Arabidopsis, the *ZoHSF* members in ginger might have undergone replication events.

The expansion of gene families in plant genomes is often attributed to genome replication, tandem replication, and fragment replication, which are considered to be the main drivers of evolution, leading to the emergence of new functions and expression patterns [44]. Previous studies have shown that replication events in certain large gene families, like WRKY, are primarily due to fragment replication and tandem duplication, while gene families, such as MADS and NBS, primarily expand through transposable replication [45]. In the case of ginger, synteny analysis confirmed that the expansion of the *ZoHSF* gene family primarily resulted from fragment duplication rather than tandem duplication, and a similar expansion occurred in the *HSF* gene family of Tartary buckwheat [46]. Furthermore, the expression analysis revealed that some duplicated *ZoHSF* genes exhibited different expression patterns in various tissues and organs. For instance, *ZoHSF16* and *ZoHSF25* are a pair of duplicated genes, but their expression patterns differ. *ZoHSF16* showed high expression levels in mature flowers and the first internode, whereas *ZoHSF25* was highly expressed in the root. The specific or redundant cellular functions of these genes during the development of other plants have been observed [47]. Subsequent investigations revealed that the motif positions and compositions of these genes were identical, suggesting that differential gene expression may be attributed to gene mutations that occurred during gene duplication, leading to the loss of certain gene segments [48]. Furthermore, changes in the motif composition during the process of gene replication may also contribute to functional differences [49]. For instance, a comparison of the motif composition between *ZoHSF01* and *ZoHSF03* highlighted differences, as *ZoHSF01* contained motif 2, motif 4, motif 1, and motif 3, whereas *ZoHSF03* lacked motif 2 (Figure 3). This discrepancy in motif composition correlated with divergent expression patterns, with *ZoHSF01* showing high expression levels in roots and *ZoHSF03* being highly expressed in flowers (Figure 8). To summarize, the variation in gene expression within gene pairs could be attributed to gene mutations or changes in motif composition during gene replication [50].

Monocotyledonous plants include *Oryza sativa*, *Musa acuminata*, *Zingiber officinale*, and *Triticum aestivum*, whereas Arabidopsis belongs to the dicotyledonous category. Analysis of the phylogenetic tree indicated that most subgroups of *ZoHSF* contained both monocotyledonous and dicotyledonous representatives (Figure 4). This suggests that *HSF* genes emerged in both types of plants prior to their differentiation [51]. Additionally, a conserved motif analysis revealed that genes within the same group in ginger shared similar sequence structures. For example, HSFA exclusively possessed motif 8 and motif 9, whereas HSFB contained motif 5; motif 3 was present in both HSFA and HSFB. Moreover, motif 1 and motif 4 were found in all HSF proteins across different groups. This specific arrangement of motifs likely contributes to the distinct functionalities exhibited by each group [52]. Similar findings have been reported in *HSF* members of other plants, such as *Phyllostachys edulis* [53], *Cucurbita moschata* [54], and *Camellia sinensis* [55].

In plants, introns play a crucial role in evolution and can be gained or lost throughout evolutionary history [56]. Consequently, analyzing gene structures is essential for understanding gene functions. In this study, we examined the gene structure of *ZoHSF* genes. Figure 3C illustrates that 40% (10/25) of *ZoHSF* genes lacked introns, while the remaining genes had one to two introns. Interestingly, genes with and without introns were evenly distributed among the three subgroups of the ginger *HSF* gene family. This phenomenon of intron-less genes has also been observed in other gene families, such as the AP2/ERF gene family and the small GRAS gene family [57]. It is noteworthy that intron-less genes may enable rapid responses to stress and regulation of plant growth and development [58]. The absence of introns in some ginger *HSF* genes suggests a potential mechanism involving horizontal gene transfer and replication of *HSF* genes during the evolution originating from nuclear genes [59].

The expressions of metabolic pathway-related genes are regulated by cis-elements in the promoter regions of gene family members [60]. Earlier research has suggested that plant hormones, including abscisic acid (ABA), jasmonic acid (JA), ethylene (Et), and salicylic acid (SA), play a crucial role in modulating the expression of HSFs during abiotic stresses, like high temperature and drought [61,62,63]. During the evaluation of cis-acting elements in the promoter region of *ZoHSFs*, we observed the presence of diverse elements, such as defense responsiveness elements, low-temperature responsiveness elements, and ABA-responsive elements (Figure 3D). This indicates that *ZoHSF* genes not only respond to different stress stimuli but also participate in the regulation of ginger growth and development through the modulation of plant hormone levels [64]. Ginger plants typically have a long flowering cycle, often taking up to 10 years to bloom. The precise mechanism underlying this phenomenon remains unclear. However, our study revealed significant expression of *ZoHSF09* in all developmental stages of ginger flowers, including the flower bud, young flower, and mature flower. This finding suggests that *ZoHSF09* may be involved in ginger flower development. Another similar gene, *ZoHSF02*, also shows potential as a candidate gene for regulating ginger flower development. Nevertheless, further experimental verification is necessary to confirm these findings. The rhizome serves as the economic organ of ginger and selecting seed ginger with robust rhizome buds for planting is an effective method to achieve high ginger yields [65]. Based on the expression pattern of *ZoHSF01*, we observed relatively high expression levels in rhizome buds, the first internode, and the secondary internodes, indicating its potential role in the process of rhizome enlargement. Notably, the expression level of *ZoHSF01* was significantly reduced in the third internode of ginger. Thus, further investigations are required to determine whether *ZoHSF01* regulates the growth and development of rhizomes.

The similarity of gene function within branches of a multi-species phylogenetic tree has been verified [66]. By analyzing the phylogenetic relationships and collinear analysis of ginger and four other plant *HSF* members, we can make initial predictions about the function of *ZoHSF* members in response to abiotic stress. Figure 4 shows that *ZoHSF16* and *ZoHSF25* are orthologous to *AtHSFA1b*, as they form a cluster. Previous studies have established that *HSFA1b* has the ability to enhance the antioxidant defense system of plants by modulating the expression of antioxidant enzymes. This, in turn, mitigates the detrimental effects of oxidative stress on plants [67]. Moreover, *HSFA1b* also plays a crucial role in regulating the expression of membrane proteins, thereby contributing to the maintenance of cell membrane integrity [68]. Such regulatory mechanisms ensure the stability of both internal and external cell environments, enabling plants to effectively combat drought stress. Consequently, it can be inferred that *ZoHSF16* and *ZoHSF25* might assist ginger in its resilience against drought stress by controlling the expression of membrane protein, thus preserving cell membrane integrity even under high-temperature conditions. Additionally, qRT–PCR analysis revealed that the expression of *ZoHSF16* and *ZoHSF25* genes is upregulated in high-temperature and strong-light stress conditions, suggesting their potential involvement in the response to these stressors in ginger. Furthermore, studies have demonstrated that *HSFA1d*, known for its role in the transcriptional regulation of *HSFA2*, serves as a critical regulator within the HSF signaling network during environmental stress [69]. Notably, when *HSFA1d* is overexpressed in cucumber, it can trigger the biosynthesis and signal transmission of endogenous jasmonic acid (JA), resulting in enhanced cold tolerance in cucumber [70]. In our study, we observed that *AtHSFA1d* and *ZoHSF11* clustered together within the same subfamily, implying that they might be orthologous and potentially share similar functions. Interestingly, several stress-related cis-acting elements, including MEJA, ABRE, and LTR, were identified in the promoter of *ZoHSF11*. Additionally, under high-temperature and strong-light stress conditions, the transcript levels of *ZoHSF11* exhibited a substantial increase, indicating its potential role in the stress response of ginger. However, additional experiments are necessary to validate the functions of the aforementioned *ZoHSFs*.

## 4. Materials and Methods

### 4.1. Plant Materials

In this study, we obtained a locally grown ginger variety called ‘Zhugen ginger’ from the Chongqing University of Arts and Sciences in Chongqing, China. To simulate the natural conditions in Chongqing, where the highest temperature can exceed 40 °C and the light intensity can reach 103,833 Lux, we exposed two-month-old ginger seedlings to an outdoor environment. The functional leaves of ginger, specifically the third to fifth unfolded leaves from the top to base stem, were collected at 8:30 a.m. and 3:00 p.m. on the first day and at 3:00 p.m. on the second, third, and fourth days. The gathered samples were promptly frozen with the use of liquid nitrogen, followed by preservation at a temperature of −80 °C for subsequent analysis. To ensure accuracy, each sample was prepared with three technical replicates.

### 4.2. Identification and Physicochemical Properties Analysis

The genomic data, including genes, cDNAs, and proteins, of *Z. officinale*, were obtained from our ginger genome research project [37]. To identify potential candidate genes of the HSF protein in ginger, the HMM data for the HSF protein domain (PF00447) were retrieved from the Pfam website (https://pfam.xfam.org/, accessed on 16 October 2022). A search using the HMM model was conducted on the *Z. officinale* genome data, and the *HSF* candidate genes were selected using a threshold value of e < 10^−5^. To validate the identified candidates, the NCBI BatchCDD search was employed. The physical and chemical properties of the newly identified *ZoHSF* members were analyzed using ProtParam (https://web.expasy.org/protparam/, accessed on 16 October 2022). The positions of the *ZoHSF* members were then mapped onto the reference genome and named according to their chromosome positions using TBtools [71].

### 4.3. Phylogenetic Analysis

To align the full-length protein sequences of reported *HSF* in *A. thaliana* and the newly identified *ZoHSFs*, MAFFT was utilized with the default parameters [72]. The alignment results were then used to construct a neighbor-joining (NJ) tree using MEGA X. The parameters for constructing the NJ tree were set as follows: Poisson model, pairwise deletion, and 1000 replicates for bootstrap values [73]. Additionally, a multi-species phylogenetic evolutionary tree was built, incorporating protein sequences of *ZoHSF*, *A. thaliana*, *T. aestivum*, *O. sativa*, and *M. acuminata HSFs* obtained from the UniProt database (https://www.uniprot.org/, accessed on 16 October 2022).

### 4.4. Genetic Structure, Motifs Composition, Gene Duplication and Cis-Acting Elements

The exon–intron structure of the *ZoHSF* genes was determined using the Gene Structure Display Server (GSDS: http://gsds.cbi.pku.edu.cn/, accessed on 17 October 2022). To identify conserved motifs within the ZoHSF proteins, we employed the MEME online tool available at http:/meme.nbcr.net/meme/intro.html (accessed on 17 October 2022). Furthermore, the plantCARE software (https://bioinformatics.psb.ugent.be/webtools/plantcare/html/, accessed on 17 October 2022) was utilized to predict the cis-acting elements within the promoters of *ZoHSFs*. An analysis of gene duplication events in ginger *HSF* members was conducted using the multiple collinear scanning toolkits (MCScanX), while the Dual Synteny software revealed the homologous relationship between the ginger *HSF* gene and those of *A. thaliana*, *T. aestivum*, *O. sativa*, and *M. acuminata*.

### 4.5. Gene Expression and qRT–PCR Analysis

In a previous study conducted by our research group, we conducted transcriptome analysis of ginger in various tissues subjected to abiotic stresses, including cold, heat, high temperature, and drought [44]. Among the samples collected, various tissues of ginger were included, such as 6-month-old flowers, buds, pedicels, stems, rhizome buds, and rhizomes. Additionally, the first, second, and third internodes, functional leaves (the third leaf from the top to the base of the stem), leaf buds, and roots were also included. For the drought and salinity treatments, the plants were irrigated with a solution containing 15% PEG6000 to induce drought stress and 200 mM NaCl for salinity stress. Heat stress treatment involved subjecting the ginger plantlets to 40 °C, while cold stress treatment involved exposing them to 4 °C. Following these treatments, leaf samples were collected at specific time points, including 0, 1, 3, 6, 12, 24, and 48 h after cold, drought, and salt treatments. Additionally, leaves were collected at corresponding time intervals of 0, 1, 3, 6, 12, and 24 h following the heat treatment. This analysis allowed us to investigate the expression patterns of *ZoHSF* members in different tissues and under abiotic stress conditions. To visualize these expression patterns, we utilized the HeatMap program of the TBtools software. The TRIzol kit (Invitrogen, California, USA) was used for the extraction of total RNA from all samples, and cDNA was synthesized from RNA using the Evo M-MLV reverse transcription kit (Accurate Biology, Shanghai, China). Primers for *ZoHSF* members (additional file 2: Table S3) were designed using the Primer premier 5.0 software. The selected *ZoHSF* genes were subjected to qRT–PCR to analyze their response to abiotic stress. The *ZoTUB2* (ZOFF_005593) gene served as an internal control, and each qRT–PCR experiment was performed in triplicate using the CFX96 Real-Time System (Bio-Rad). The following protocol was followed: an initial denaturation step at 95 °C for 30 s, followed by a series of 40 cycles, each consisting of denaturation at 95 °C for 10 s and annealing at 60 °C for 30 s. To ensure biological accuracy, each reaction was performed three times as a replicate. The expression data were quantified using the 2^−(ΔΔCt)^ method [74].

## 5. Conclusions

In conclusion, we identified a total of 25 members of the *ZoHSF* gene family in the ginger genome, and they exhibited uneven distribution across ten chromosomes. These *ZoHSF* members were classified into three groups, namely HSFA, HSFB, and HSFC. Additionally, our analysis revealed that 12 *ZoHSF* genes likely originated from duplication events. Through promoter analysis, it was observed that hormone response elements, such as MEJA responsiveness and abscisic acid responsiveness, were prevalent among the cis-elements associated with abiotic stress response in *ZoHSF* promoters. Furthermore, by examining the phylogenetic and collinear relationships between ginger and other plant HSF members, we gained preliminary insights into the function of various *ZoHSF* genes. Specifically, our findings showed that *ZoHSF16* and *ZoHSF25* are orthologous to *AtHSFA1b* and likely play a role in ginger’s response to high temperature and strong light stress. The duplicated gene pairs *ZoHSF16* and *ZoHSF25* suggest functional similarities between them. Moreover, our results suggest that *ZoHSF11*, which shares orthology with *AtHSFA1d*, may be involved in ginger’s stress response process. This is supported by its increased expression under high-temperature and strong-light stress conditions, as well as the presence of stress-related cis-acting elements on its promoter. Overall, this research enhances our understanding of the molecular mechanisms underlying stress responses in ginger plants and paves the way for further investigation of *HSF* genes in this species.

## Figures and Tables

**Table 1:** List of the 25 *ZoHSF* genes identified in this study.

Gene	Gene ID	Chromosome	Stand	Start	End	a.a. Length	Mw (KDa)	pI
*ZoHSF01*	Maker00046875	Chr02	+	91065794	91067203	266	30.07	9.01
*ZoHSF02*	Maker00044356	Chr04	+	33998421	34000016	270	29.54	9.62
*ZoHSF03*	Maker00044280	Chr04	−	96993139	97002690	499	56.5	9.56
*ZoHSF04*	Maker00071535	Chr06	+	24787075	24788896	403	46.48	5.43
*ZoHSF05*	Maker00055135	Chr06	−	133330245	133331293	308	34.35	5.32
*ZoHSF06*	Maker00011550	Chr06	−	139482237	139484877	433	49.38	5.06
*ZoHSF07*	Maker00069644	Chr08	+	22713092	22719084	585	64.4	5.87
*ZoHSF08*	Maker00075123	Chr08	−	59803301	59805080	348	39.79	5.15
*ZoHSF09*	Maker00055735	Chr08	−	106764803	106766617	473	52.22	4.7
*ZoHSF10*	Maker00015929	Chr08	+	114492344	114493480	350	38.04	7.17
*ZoHSF11*	Maker00022825	Chr12	−	8840276	8843152	471	52.64	5.99
*ZoHSF12*	Maker00077492	Chr12	+	26467862	26473127	482	53.7	4.83
*ZoHSF13*	Maker00077650	Chr12	+	41429765	41431199	350	38.63	5.19
*ZoHSF14*	Maker00078166	Chr12	+	43443734	43444720	239	27.65	9.22
*ZoHSF15*	Maker00051334	Chr12	+	96546341	96550898	448	49.91	5.29
*ZoHSF16*	Maker00051145	Chr14	−	101188075	101193908	484	54.07	4.97
*ZoHSF17*	Maker00046223	Chr16	+	42933888	42937462	520	57.55	4.62
*ZoHSF18*	Maker00029361	Chr18	+	79243250	79245390	336	39.03	5.11
*ZoHSF19*	Maker00022535	Chr20	+	4635869	4636990	287	31.86	8.49
*ZoHSF20*	Maker00060698	Chr20	−	46787370	46789444	329	38.18	4.92
*ZoHSF21*	Maker00067853	Chr20	+	57334656	57336116	260	30.11	7.86
*ZoHSF22*	Maker00068042	Chr20	−	63455337	63456430	336	36.96	5.36
*ZoHSF23*	Maker00000351	Chr20	−	154043307	154044447	350	38.11	5.9
*ZoHSF24*	Maker00008530	Chr22	+	121498523	121499557	319	35.44	6.9
*ZoHSF25*	Maker00008307	Chr22	+	124703631	124709234	498	55.65	5.06

## Data Availability

The data utilized in this study are currently not publicly available as they are part of the ginger genome and have not been released. However, the data presented in this study can be made available upon request from the corresponding author.

## Supplementary Materials

The following supporting information can be downloaded at https://www.mdpi.com/article/10.3390/plants12162999/s1, additional file 1: Table S1: Analysis and distribution of conserved motifs in other four plants HSF proteins. Table S2: Analysis and distribution of conserved motifs in *Zingiber officinale* HSF proteins; additional file 2: Table S3: The primer sequences for qRT-PCR used in this study.
